# Integrated Transcriptomic and Functional Analyses Identify an FSH-Regulated circARHGAP21/gga-miR-146a-5p/*DEAF1* Module in Chicken Granulosa Cells

**DOI:** 10.3390/cells15211939

**Published:** 2026-10-25

**Authors:** Yu Ou, Pucheng Wu, Bingkun Xu, Sheng Wang, Shengxiao Cao, Yuhan Sun, Zixin Zhang, Jinshuo Song, Xue Sun, Ning Qin, Rifu Xu

**Affiliations:** 1 College of Animal Science and Technology, Jilin Agricultural University, Changchun 130118, China; ouyu12380@mails.jlau.edu.cn (Y.O.); w1602859841@163.com (P.W.); xbkingbk@163.com (B.X.); haohunzai@163.com (S.W.); jncsxiao@mails.jlau.edu.cn (S.C.); sunyh990406@163.com (Y.S.); zxinhhhh@163.com (Z.Z.); songjinshuo2026@163.com (J.S.); xuesun1128@163.com (X.S.); 2 Key Lab of Animal Production, Product Quality and Security, Ministry of Education, Jilin Agricultural University, Changchun 130118, China

**Keywords:** granulosa cells, FSH, follicle selection, circARHGAP21, gga-miR-146a-5p, *DEAF1*

## Abstract

Follicle-stimulating hormone (FSH) supports granulosa cell survival during follicular development, but the RNA networks involved remain incompletely understood. To investigate these networks, we integrated whole-transcriptome profiling and functional assays in cultured granulosa cells from chicken prehierarchical follicles. Transcriptomic comparisons included control, FSH and FSH plus leptomycin B (LMB), a nuclear export inhibitor. Integrated analysis of these profiles identified a candidate circARHGAP21/gga-miR-146a-5p/*DEAF1* regulatory module. FSH increased circARHGAP21 and *DEAF1* expression and decreased gga-miR-146a-5p abundance, whereas LMB opposed these changes in FSH-treated cells. Reporter assays showed that gga-miR-146a-5p suppressed reporter activity through the predicted sites in circARHGAP21 and the *DEAF1* 3′ untranslated region. Transfection with the oe-circARHGAP21 construct reduced apoptosis and increased follicle-stimulating hormone receptor (*FSHR*) expression. Construct transfection also increased proliferating cell nuclear antigen (PCNA) expression and decreased caspase-3 (CASP3) expression. Cotransfection with a gga-miR-146a-5p mimic attenuated these effects, whereas *DEAF1* overexpression produced changes similar to those observed after oe-circARHGAP21 construct transfection. Compared with the oe-circARHGAP21 construct group, the corresponding LMB-treated group showed lower *DEAF1*, *FSHR* and *PCNA* expression and higher *CASP3* expression and apoptosis. Together, these findings identify an FSH-responsive circARHGAP21/gga-miR-146a-5p/*DEAF1* module associated with granulosa cell survival and follicle-selection-related gene expression.

## 1. Introduction

Follicle selection determines whether a prehierarchical follicle enters the preovulatory hierarchy in hens, thereby sustaining ovulation and egg production [1,2,3]. During this transition, granulosa cells (GCs) acquire FSH responsiveness and coordinate survival, proliferation and differentiation [1,2,4]. FSH binding to FSHR activates cAMP/PKA and PI3K/AKT signaling, inducing receptor expression and steroidogenic programs while suppressing apoptosis [4,5,6,7,8,9,10]. Expression of the follicle-stimulating hormone receptor gene *FSHR* increases as hen GCs acquire selection-associated FSH responsiveness, making *FSHR* a molecular marker of follicle selection [11]. Proliferating cell nuclear antigen (PCNA) is a DNA-replication-associated marker of proliferative activity, whereas caspase-3 (CASP3) is a central executioner of apoptosis [12,13]. However, how FSH signaling cooperates with subcellular transport and post-transcriptional regulation to stabilize a selection-associated GC state remains poorly understood.

Nucleocytoplasmic transport provides a regulatory interface between extracellular signals and gene expression. The nuclear pore complex selectively controls the bidirectional transport of proteins and ribonucleoprotein complexes between the nucleus and cytoplasm [14,15]. Chromosome region maintenance 1 (CRM1), also known as exportin 1 or XPO1, recognizes leucine-rich nuclear export signals in a RanGTP-dependent manner [16,17]. The canonical CRM1 inhibitor leptomycin B (LMB) blocks cargo binding through the covalent modification of CRM1 and is widely used to perturb nuclear export [18,19]. In chicken GCs, FSH-activated AKT promotes FOXO1 phosphorylation and nuclear exclusion [7]. FOXO1 also contributes to GC apoptosis and cell-cycle inhibition in mammalian models [10]. We previously linked FSH-induced FOXO1 nuclear exclusion to *FSHR* expression, proliferation and survival in chicken GCs [7]. These findings implicate nuclear transport in follicle selection, but its effects on FSH-responsive RNA networks remain unclear.

FSH-responsive changes in gene expression extend beyond mRNAs to other RNA classes. MicroRNAs regulate target mRNA stability and translation [20,21]. CircRNAs are generated via back-splicing and can influence RNA stability, transcription, translation and RNA-binding protein interactions [22,23,24,25,26,27,28]. In chicken ovarian GCs, circRNA expression changes across follicular development [29]. Ovarian miRNAs regulate post-transcriptional gene expression, GC apoptosis, follicular development and steroidogenesis [30,31,32,33]. miR-146a-5p participates in diverse regulatory programs in other cell types [34,35]. In chicken GCs, it attenuates AKT signaling, proliferation, lipid deposition and progesterone biosynthesis [36]. Some back-spliced circRNAs can participate in circRNA–miRNA regulation, but a mechanistic interpretation requires evidence for colocalization, stoichiometry and endogenous target derepression [37,38,39,40,41]. It remains unknown which non-coding RNAs respond to FSH in chicken GCs, how these responses are altered by nuclear-export perturbation and whether the resulting candidate modules are associated with apoptosis.

Here, we compared mRNA, miRNA and circRNA profiles in cultured GCs from 6–8 mm chicken prehierarchical follicles. Cells were assigned to control, FSH or FSH plus LMB conditions. LMB served as a pharmacological screening tool to prioritize FSH-regulated RNA networks containing proteins with potential nucleocytoplasmic shuttling functions. We combined differential expression analysis, nine-quadrant screening for opposing responses and target prediction to select the circARHGAP21/gga-miR-146a-5p/*DEAF1* candidate module. We used qRT-PCR, RNase R digestion, localization and reporter assays, overexpression and miRNA-mimic cotransfection to assess molecular characteristics and associations with FSHR, PCNA, CASP3 and apoptosis.

## 2. Materials and Methods

### 2.1. Animals and Ethical Approval

All animal procedures were approved by the Animal Care and Use Committee of Jilin Agricultural University (approval no. GR(J)19-030; 1 April 2023). Hens underwent ether anesthesia followed by carotid artery exsanguination before tissue collection, according to the approved protocol, with measures taken to minimize suffering.

### 2.2. Collection of Prehierarchical Follicles and GC Isolation and Culture

A source flock of 80 21-week-old Lohmann Brown laying hens was maintained under uniform husbandry conditions at the Jilin Agricultural University breeding base. Hens were housed individually with ad libitum feed and water under a 16 h light/8 h dark photoperiod. Husbandry followed our previously reported procedures [7,42,43]. Of this flock, 21 hens served as tissue donors: 9 were used exclusively for sequencing (3 per treatment group), and 12 provided material for non-sequencing assays. Within the non-sequencing cohort, donor material was shared between qRT-PCR and transfection-based assays and between Western blotting and immunofluorescence; reuse across assays did not constitute additional biological replicates. The remaining 59 hens continued to be maintained in the source flock under routine husbandry and underwent no experimental procedures for this study. These 21 hens were euthanized, and the ovaries were immediately placed in ice-cold phosphate-buffered saline (PBS; Biosharp, Hefei, China). Approximately 10 prehierarchical follicles measuring 6–8 mm were collected aseptically per hen and washed twice in antibiotic-containing PBS. After removal of the yolk and surrounding connective tissue, the granulosa layer was isolated and minced. It was then digested with 0.2% type II collagenase (Biosharp) for 15 min at 37 °C. Digestion was terminated with an equal volume of complete M199 medium. The suspension was filtered through a 200-mesh cell strainer and centrifuged at 1200 rpm for 5 min. Cells were washed three times with PBS and resuspended in M199 containing 10% fetal bovine serum (Procell, Wuhan, China), 100 IU/mL penicillin and 75 IU/mL streptomycin. Cells were seeded in six-well plates at approximately 5 × 10^6^ cells per well and cultured at 37 °C with 5% CO_2_ under saturated humidity. The medium was replaced after a 12 h attachment period, before treatment.

### 2.3. FSH and LMB Treatments

FSH treatment conditions were based on previous work [7,44], whereas the LMB condition followed our earlier study [7]. Attached GCs were assigned to three groups. LMB was dissolved in dimethyl sulfoxide (DMSO), and the final DMSO concentration was matched across conditions. Control cells (C) received the corresponding vehicle. FSH cells (F) received 10 ng/mL FSH (Ningbo Second Hormone Factory, Ningbo, China). FSH plus LMB cells (L) received 10 ng/mL FSH and 10 ng/mL LMB (Beyotime, Shanghai, China). All treatments lasted 12 h and included three biological replicates. Each biological replicate was processed separately for cell preparation, treatment and RNA extraction. Cells from the sequencing cohort were collected for whole-transcriptome analysis; molecular validation used the non-sequencing cohort.

### 2.4. Total RNA Extraction, Quality Control and Sequencing Samples

Total RNA was extracted from treated GCs using a total RNA isolation reagent (Biosharp, Hefei, China) according to the manufacturer’s instructions. Cells were washed twice with ice-cold PBS before lysis. After phase separation, precipitation and washing, RNA was dissolved in RNase-free water. The concentration and purity were measured spectrophotometrically, and samples with OD260/OD280 ratios of 1.8–2.1 underwent further assessment. RNA degradation and genomic DNA contamination were evaluated via 1% agarose gel electrophoresis. Integrity was assessed using an Agilent 2100 Bioanalyzer (Agilent Technologies, Santa Clara, CA, USA). Samples with an RNA integrity number of at least 7.0 were used for library preparation. Three biological replicates per group yielded nine total RNA samples for long- and small-RNA library preparation. The nine samples were processed together for RNA extraction and, within each library type, library preparation and sequencing.

### 2.5. Whole-Transcriptome Library Preparation and Sequencing

Strand-specific mRNA/circRNA libraries were prepared after ribosomal RNA depletion. Enriched RNA was fragmented and reverse-transcribed using random primers, with dUTP incorporated during second-strand cDNA synthesis. Strand-specific libraries were generated via end repair, A-tailing, adaptor ligation, uracil-N-glycosylase digestion, PCR amplification and size selection. Gene Denovo Biotechnology Co., Ltd. (Guangzhou, China) sequenced these libraries on an Illumina HiSeq 4000 platform (Illumina, San Diego, CA, USA) using paired-end sequencing. For small-RNA libraries, 18–30 nt RNA fragments were recovered via polyacrylamide gel electrophoresis. The 3′ and 5′ adaptors were ligated sequentially, followed by reverse transcription and PCR. Approximately 140 bp products were purified and assessed using an Agilent 2100 Bioanalyzer and qPCR. Gene Denovo Biotechnology Co., Ltd. sequenced the libraries on an Illumina NovaSeq 6000 platform.

### 2.6. mRNA, miRNA and circRNA Expression Profiling

Raw sequencing data were subjected to quality control using fastp v0.18.0 [45]. For mRNA/circRNA data, reads with adaptor contamination or more than 10% ambiguous bases were removed. Poly(A)-only reads and reads with over 50% of bases at quality scores of 20 or lower were also discarded. For small-RNA data, adaptor-contaminated, low-quality and insert-free reads were removed. Reads with inserts shorter than 18 nt or poly(A) contamination were also excluded. Filtered clean reads or clean tags were used for downstream analyses.

For mRNA and circRNA analyses, clean reads were first aligned to an rRNA database using Bowtie2 v2.2.8 [46]. The remaining reads were aligned to the *Gallus gallus* reference genome using HISAT2 [47]. Unmapped reads were used to identify circRNAs. Briefly, 20 nt anchors from both read ends were realigned to identify back-splice junctions. CIRIquant v1.1.3 was used for circRNA identification. Candidate circRNAs required at least two unique back-splice junction reads in at least one sample. circRNA abundance was normalized as back-splice junction reads per million total back-splice junction reads (RPM). Differential expression was analyzed using edgeR v3.12.1 [48], with a |log2 fold change| ≥ 1 and nominal *p* < 0.05. Because this analysis was intended for candidate discovery, unvalidated circRNA changes were interpreted as exploratory.

For miRNA analyses, clean tags were aligned to GenBank release 209.0 and Rfam [49]. rRNA, tRNA, snRNA and other non-miRNA small RNAs were removed. The remaining tags were aligned to miRBase release 22 to identify known miRNAs [50]. miRDeep2 [51] predicted novel miRNAs from unannotated tags using genomic positions, precursor hairpins and read features. miRNA abundance was normalized as transcripts per million. Differential expression was analyzed using edgeR, with a |log2 fold change| ≥ 1 and nominal *p* < 0.05.

For mRNA analyses, StringTie v1.3.1 [52] assembled and quantified transcripts from HISAT2 alignments. Differential expression was assessed using DESeq2 [53]. Genes were classified as differentially expressed at a false discovery rate of 0.05 or lower and |log2 fold change| ≥ 1. Low read counts and small replicate numbers can limit the differential expression detection power after multiple-testing adjustment [53]. To retain candidates for follow-up, nominal-*P* miRNA/circRNA screening was used for exploratory prioritization, with adjusted values retained in Supplementary Table S2. The prioritized module was subsequently assessed via qRT-PCR and targeted functional assays. Unless otherwise stated, bioinformatic analyses used three biological replicates per group as the statistical units.

### 2.7. Functional Classification, Pathway Enrichment, Nine-Quadrant Screening and Candidate ceRNA Network Construction

GO level-2 functional classification summarized the distribution of differentially expressed and nine-quadrant-selected mRNAs across functional categories. KEGG pathway enrichment was assessed using a hypergeometric test. A nominal *p* < 0.05 indicated exploratory enrichment, whereas *Q* ≤ 0.05 indicated enrichment supported after multiple-testing correction. Supplementary Table S6 provides GO category counts and gene lists, together with KEGG pathway labels, gene counts, background sizes and the original *P* and *Q* values.

Nine-quadrant analysis integrated differential expression in the F-versus-C and L-versus-F comparisons, labeled C/F and F/L, respectively. The vertical axis shows C/F (F relative to C), and the horizontal axis shows F/L (L relative to F). Upregulation indicates higher expression in F than C for C/F and in L than F for F/L. Candidates showing opposing expression changes in the two comparisons were prioritized. These included molecules increased by FSH and decreased after LMB addition or decreased by FSH and increased after LMB addition. The selected mRNAs, miRNAs and circRNAs were used for network construction and experimental characterization.

miRNA–mRNA interactions were predicted using miRanda v3.3a [54] and TargetScan release 7.0 [55]. Targets predicted by both algorithms were retained as the predicted target set. circRNA–miRNA interactions were predicted using miRanda. Correlations were calculated across all nine transcriptome samples without treatment-group adjustments. miRNA–mRNA and miRNA–circRNA pairs required predicted targeting and a Spearman coefficient < −0.7. Candidate circRNA–mRNA pairs required a Pearson coefficient > 0.9 and the enrichment of shared miRNAs based on hypergeometric testing (nominal *p* < 0.05). Molecules meeting these criteria were assembled into a circRNA–miRNA–mRNA candidate network using Cytoscape v3.10.2 [56].

### 2.8. Reverse Transcription and Quantitative Real-Time PCR

For mRNA and circRNA measurements, total RNA was reverse-transcribed using a kit from Tolobio (Shanghai, China). Reactions were incubated at 50 °C for 15 min and then at 85 °C for 5 s. miRNAs were reverse-transcribed using a Tolobio stem-loop miRNA kit. qRT-PCR was performed using SYBR Green qPCR Mix (Tolobio). Each 20 μL reaction contained 10 μL of 2× SYBR mix, 0.4 μL of each primer, 2 μL of cDNA and 7.2 μL of nuclease-free water. Cycling comprised 95 °C for 10 min, followed by 40 cycles of 95 °C for 15 s and 60 °C for 60 s. Melt-curve analysis was performed after amplification. Each biological replicate included three technical wells and a no-template control. *β-actin*, *U6* and *GAPDH* served as reference genes for mRNA, miRNA and circRNA measurements, respectively. circARHGAP21 was measured using a primer designed to target the annotated back-splice junction, whereas linear *ARHGAP21* used a transcript-specific primer. Relative expression was calculated using the 2^−ΔΔCt^ method. Primer sequences are listed in Table 1.

### 2.9. RNase R Digestion

To assess the RNase R resistance of circARHGAP21, total RNA from FSH-treated GCs was divided equally between RNase R-treated and untreated groups. The untreated aliquot received an equal volume of RNase-free water. The 20 μL reaction contained <5 μg RNA, 2 μL of 10× buffer and RNase R at 1–3 U/μg RNA (Supplementary Table S4). RNase R (Beyotime, Shanghai, China) digestion proceeded at 37 °C for 20 min, followed by inactivation at 70 °C for 10 min. Equal volumes of the treated and untreated RNA were reverse-transcribed. Relative circARHGAP21 and linear *ARHGAP21* abundance was measured via qRT-PCR, using *GAPDH* from the matched untreated aliquot as the common reference for both aliquots. The annotated circARHGAP21 sequence is provided in Supplementary Table S5.

### 2.10. RNA Fluorescence In Situ Hybridization and Immunofluorescence

RNA fluorescence in situ hybridization (RNA-FISH) was performed to determine the subcellular distributions of circARHGAP21 and gga-miR-146a-5p. GCs were seeded on coverslips, fixed for 5 min in acetone pre-cooled to −20 °C and washed three times with PBS (pH 7.4). Cells were treated with 5 μg/mL proteinase K (Servicebio, Wuhan, China) at 40 °C and pre-hybridized for 1 h. Target-specific probes were then hybridized overnight at 40 °C. The probe sequences and detailed hybridization conditions are provided in Table 2. After hybridization, samples were washed sequentially with 2× SSC, 1× SSC and 0.5× SSC at 40 °C. Samples were then incubated with the corresponding branch probes for 45 min and fluorescent signal probes (1:200) for 3 h at 40 °C. Nuclei were counterstained with DAPI, and coverslips were mounted with antifade mounting medium. Fluorescence images were acquired using a fluorescence microscope (Nikon, Tokyo, Japan). DEAF1 localization was examined via immunofluorescence (IF). GCs grown on coverslips were washed twice with PBS and fixed with 4% paraformaldehyde for 30 min at 4 °C. After blocking with BSA (Servicebio) for 30 min, cells were incubated with a rabbit polyclonal anti-DEAF1 antibody (Proteintech, Wuhan, China) for 12 h at 4 °C. After washing with PBS, cells were incubated with CoraLite488-conjugated goat anti-rabbit IgG (H + L) (Proteintech) at room temperature in the dark. Nuclei were counterstained with DAPI, and fluorescence images were acquired using an ECLIPSE Ci upright fluorescence microscope (Nikon, Tokyo, Japan).

### 2.11. Nucleocytoplasmic RNA Fractionation

Nuclear and cytoplasmic RNA were separated using the Cytoplasmic & Nuclear RNA Purification Kit (Norgen Biotek, Thorold, ON, Canada) according to the manufacturer’s instructions. Cells were washed with PBS, lysed and centrifuged to separate the cytoplasmic supernatant from the nuclear pellet. RNA from both fractions was purified, reverse-transcribed and analyzed via qRT-PCR.

### 2.12. Plasmid Construction, miRNA Mimic Preparation and Cell Transfection

The full annotated circARHGAP21 sequence was cloned into the pcDNA3.1(+) CircRNA Mini Vector to generate the oe-circARHGAP21 construct. The chicken *DEAF1* coding sequence was cloned into pcDNA3.1(+) to generate oe-*DEAF1*. A gga-miR-146a-5p mimic and corresponding negative control were synthesized from the mature sequence. Plasmids, the mimic and controls were designed and synthesized by Abace Biotechnology Co., Ltd. (Changsha, China). At 70–80% confluence, GCs were transfected using a transfection reagent (Biosharp, Hefei, China). The complete medium was removed, and cells were washed with PBS before the addition of serum-free medium. The plasmid or miRNA mimic and transfection reagent were separately diluted in Opti-MEM, incubated at room temperature, combined and added to the cells. The complete medium was restored after 6 h, and cells were collected after a further 24 h. Each experiment included the appropriate empty-vector or mimic-negative control. For cotransfection experiments, the gga-miR-146a-5p mimic or its control was introduced into GCs transfected with the oe-circARHGAP21 construct. LMB experiments compared molecular and apoptotic phenotypes in GCs transfected with the oe-circARHGAP21 construct with or without LMB.

### 2.13. Dual-Luciferase Reporter Assay

Dual-luciferase assays were performed in 293T cells. A circARHGAP21 fragment containing the predicted gga-miR-146a-5p site (novel_circ_010489, nt 651-900) was cloned into pmirGLO (Promega, Madison, WI, USA). A *DEAF1* 3′ UTR fragment (nt 1501-1755) was cloned separately, generating pmirGLO-novel_circ_010489-WT and pmirGLO-*DEAF1*-WT. In circARHGAP21, the wild-type sequence 5′-ATTACAGTTCTC-3′ was replaced by 5′-TAAAGTCAAGAG-3′. In the *DEAF1* 3′ UTR, 5′-CTCATGTGTGAATAAAGTTCTC-3′ was replaced by 5′-GAGTACTGTCTTAAATCAAGAG-3′, generating mutant reporters. The sense and antisense mimic strands were 5′-UGAGAACUGAAUUCCAUGGGUU-3′ and 5′-CCCAUGGAAUUCAGUUCUCAUU-3′, respectively. 293T cells were seeded in 24-well plates and cultured overnight. The medium was replaced 2 h before transfection at approximately 70% confluence. Each well received 100 ng of WT or mutant reporter and 30 nM mimic or negative control in 50 μL of Opti-MEM (Gibco, Grand Island, NY, USA). Transfection was performed using 1.5 μL of Lipofectamine 3000 (Life Technologies, Carlsbad, CA, USA). Complexes were incubated for 15–20 min at room temperature before addition. After 48 h, cells were washed twice with PBS and lysed in 100 μL of Cell Lysis Buffer. Lysates were centrifuged at 10,000–15,000 rpm for 3–5 min. A 20 μL aliquot of supernatant was assayed with 20 μL of Firefly Luciferase Assay Reagent and 20 μL of Renilla Luciferase Assay working solution. Luminescence was measured with a SpectraMax L plate reader (Molecular Devices, San Jose, CA, USA) following the Promega Dual-Luciferase protocol. Signals were acquired for 10 s at 2 s intervals. Relative reporter activity was calculated as the ratio of firefly to Renilla relative light units. Each condition included three independent replicates.

### 2.14. Western Blotting

Total protein was extracted using RIPA buffer (Biosharp, Hefei, China) containing a protease-inhibitor cocktail (Yeasen, Shanghai, China). Cells were washed two or three times with ice-cold PBS and lysed in RIPA for 30 min on ice with repeated pipetting. Lysates were centrifuged at 12,000 rpm for 10 min at 4 °C, and supernatants were collected. The protein concentration was measured by performing a BCA assay. Samples were mixed with 5× loading buffer at a ratio of 4:1 and denatured in boiling water for 15 min. Equal protein amounts were resolved via 10% SDS-PAGE at 75 V through the stacking gel and 120 V through the resolving gel. Proteins were transferred to methanol-activated PVDF membranes at 300 mA for 30 min in an ice-water bath. Membranes were blocked with 3% BSA for 30 min at room temperature and incubated with primary antibodies for 15 h at 4 °C. Antibody details are provided in Table 3. After three 5 min TBST washes, membranes were incubated with HRP-conjugated goat anti-rabbit or goat anti-mouse secondary antibodies for 30 min. Membranes were washed three more times with TBST for 5 min each before detection using an enhanced ECL kit (Beyotime, Shanghai, China; P0018M). Images were acquired using a Clinx ChemiScope 6100 system (Clinx Science Instruments, Shanghai, China). Band densities were quantified using AlphaEaseFC (Alpha Innotech, San Leandro, CA, USA). DEAF1, PCNA and CASP3 band densities were normalized to β-actin. All samples included in each comparison were processed under identical loading, transfer, antibody-incubation and exposure conditions.

### 2.15. Flow Cytometry

GC apoptosis was measured using an Annexin V-FITC/PI Apoptosis Detection Kit (MultiSciences, Hangzhou, China). Treated cells were collected using EDTA-free trypsin, centrifuged at 1500 rpm for 5 min and washed twice with PBS. Approximately 1 × 10^5^ cells were resuspended in 1× binding buffer. Cells were incubated with 5 μL Annexin V-FITC and 10 μL PI for 5 min at room temperature in the dark. Data were immediately acquired using a CytoFLEX flow cytometer (Beckman Coulter, Brea, CA, USA). Compensation was based on single-stained controls. Debris was excluded based on forward and side scatter, and aggregates were excluded using a singlet gate. Annexin V−/PI− cells were considered viable, and Annexin V+/PI− cells were considered early apoptotic. Annexin V+/PI+ cells were considered late apoptotic or secondarily necrotic, whereas Annexin V−/PI+ cells were considered necrotic. The total apoptotic fraction was calculated as the sum of early and late apoptotic fractions. Each group included three biological replicates.

### 2.16. Statistical Analysis

Statistical analyses were performed using SPSS 27.0 (IBM, Armonk, NY, USA), except for transcriptomic differential expression analysis and RNase R reanalysis. For primary-GC treatment comparisons, *n* = 3 denotes independent individual-hen biological samples per condition, with no pooling across hens or donor overlap between treatment groups. Technical replicates were not treated as independent observations. No formal randomization, blinding or a priori sample-size calculation was performed. The normality of continuous variables was assessed using the Shapiro–Wilk test, and the homogeneity of variance was also evaluated. When parametric assumptions were met, two groups were compared using a two-sided unpaired Student’s *t*-test. Three or more groups were analyzed via one-way ANOVA followed by Tukey’s post hoc test. RNase R-treated and untreated aliquots were compared using two-sided paired *t*-tests based on log2 relative expression, with a Holm adjustment across the two transcripts. Data are reported as means ± standard deviations (SDs), with replicate numbers specified in figure legends. *p* < 0.05 was considered statistically significant, and *p* < 0.01 was considered highly significant.

## 3. Results

### 3.1. FSH-Induced Transcriptome Changes in GCs Are Altered by LMB

**Figure 1:**
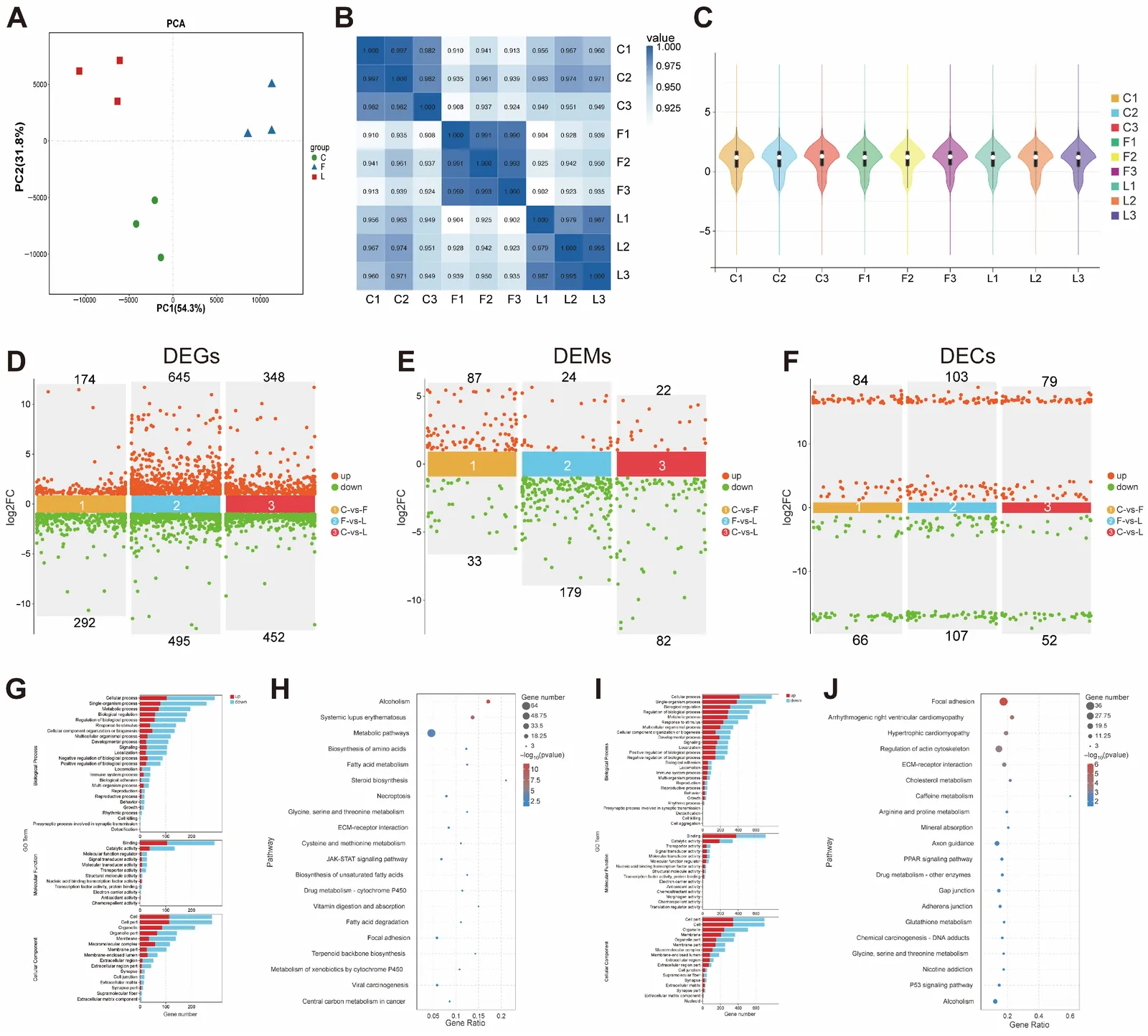
FSH-responsive transcriptome changes altered by LMB in cultured GCs from chicken prehierarchical follicles. (**A**) Principal component analysis of RNA-seq samples. (**B**) Correlations among samples. (**C**) Global expression distributions. (**D**–**F**) Numbers of mRNA DEGs (FDR ≤ 0.05) and nominal-*P* miRNA/circRNA candidates in F-versus-C, L-versus-F and L-versus-C comparisons. (**G**,**I**) GO level-2 functional classification of mRNA DEGs in the F-versus-C and L-versus-F comparisons, respectively. (**H**,**J**) KEGG pathway enrichment for the corresponding comparisons. C, control; F, FSH; L, FSH plus LMB. *n* = 3 individual-hen biological samples per group. Full GO category labels and counts and KEGG pathway labels and statistics are provided in Supplementary Table S6; KEGG enrichment supported only by nominal *p* < 0.05 is exploratory.

Whole-transcriptome sequencing was used to compare FSH-regulated expression with and without LMB and identify candidate RNA networks. GCs were assigned to control (C), FSH (F) and FSH plus LMB (L) groups. Each long-RNA sequencing sample yielded 7.29 × 10^7^–1.01 × 10^8^ raw reads and 7.27 × 10^7^–1.00 × 10^8^ clean reads after quality control. Q20 and Q30 values exceeded 96% and 90%, respectively. Total alignment rates ranged from 93.62% to 94.77%, and unique alignment rates ranged from 87.94% to 91.89% (Supplementary Table S1). Principal component analysis separated the C, F and L groups, whereas biological replicates clustered within each group (Figure 1A). Sample correlations were high within groups (Figure 1B), and global expression distributions were comparable across samples (Figure 1C).

Differential expression analysis identified differentially expressed genes (DEGs) and nominal-*P* miRNA/circRNA candidates (Figure 1D–F). The F-versus-C comparison identified 466 DEGs, including 174 upregulated and 292 downregulated genes. The L-versus-F comparison identified 1140 DEGs, including 645 upregulated and 495 downregulated genes. The L-versus-C comparison identified 800 DEGs, comprising 348 upregulated and 452 downregulated genes. The three comparisons identified 87, 24 and 22 upregulated miRNAs and 33, 179 and 82 downregulated miRNAs, respectively. The corresponding circRNA counts were 84/66, 103/107 and 79/52 (Figure 1D–F and Supplementary Table S2). The GO classification of F-versus-C DEGs highlighted cellular processes, biological regulation and metabolism (Figure 1G), while the KEGG analysis identified alcoholism and fatty acid degradation at a nominal *p* < 0.05 (Figure 1H). GO classification summarized the functional categories of L-versus-F DEGs (Figure 1I). KEGG enrichment in focal adhesion, ECM–receptor interaction and regulation of the actin cytoskeleton remained significant after multiple-testing correction (*Q* ≤ 0.05; Figure 1J). Together, these results show that FSH elicited coordinated transcriptomic changes in GCs and that LMB broadly altered the FSH-responsive expression profile.

### 3.2. Integrated Network Analysis Identifies a Candidate circARHGAP21/gga-miR-146a-5p/DEAF1 Module

**Figure 2:**
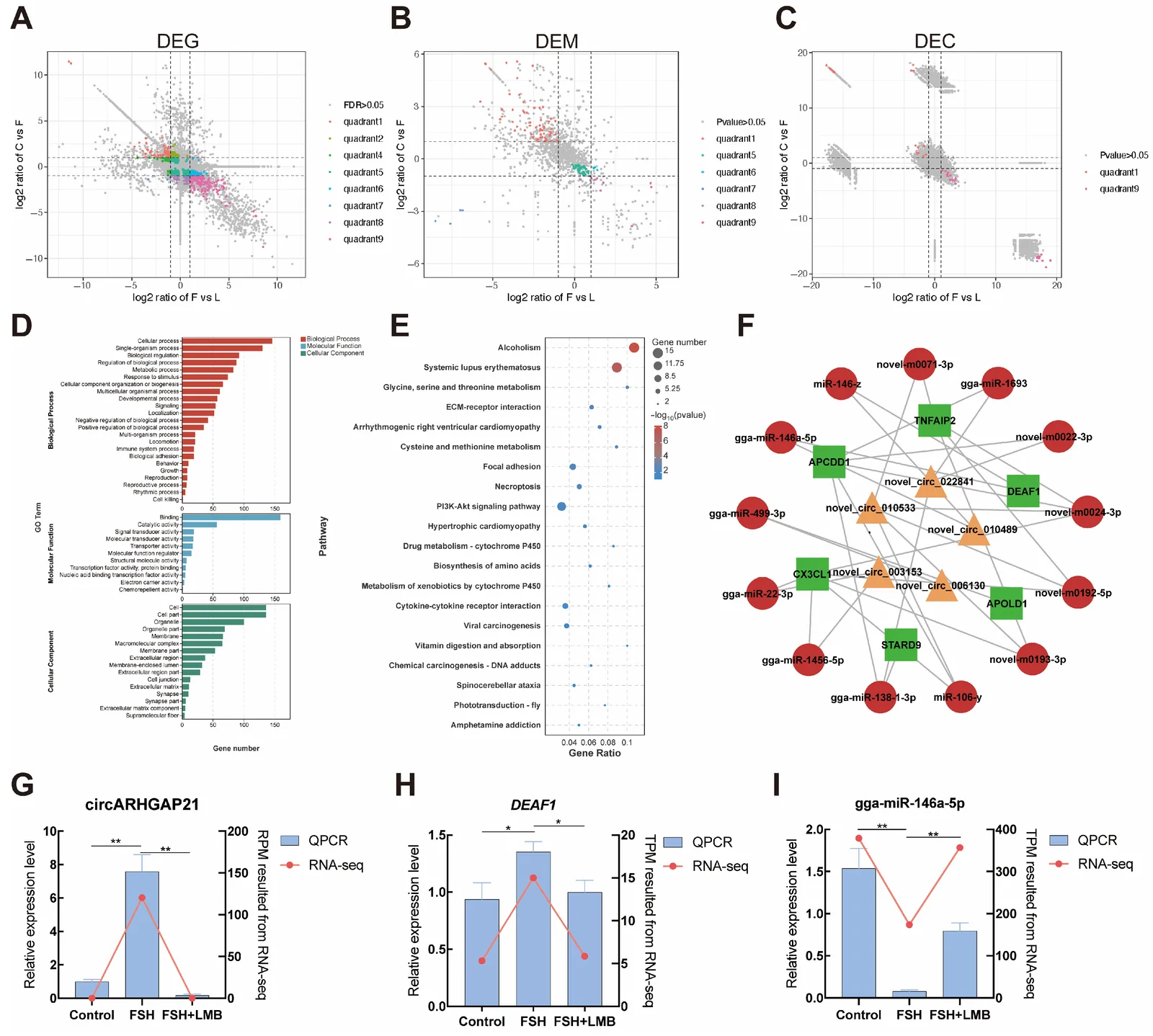
Integrated screening identifies the candidate circARHGAP21/gga-miR-146a-5p/*DEAF1* module. (**A**–**C**) Nine-quadrant screening of candidate mRNAs, miRNAs and circRNAs. The vertical axis is labeled C/F and represents F versus C; the horizontal axis is labeled F/L and represents L versus F. (**D**) GO level-2 functional classification of candidate mRNAs. (**E**) KEGG pathway enrichment of candidate mRNAs; enrichment supported only by a nominal *p* < 0.05 is exploratory, and full labels, counts and *P*/*Q* values are provided in Supplementary Table S6. (**F**) Predicted candidate circRNA–miRNA–mRNA interaction network. (**G**–**I**) qRT-PCR validation of circARHGAP21, *DEAF1* and gga-miR-146a-5p expression. Data are means ± SDs from three individual-hen biological replicates per condition. * *p* < 0.05, ** *p* < 0.01.

Nine-quadrant analysis integrated the F-versus-C and L-versus-F comparisons to identify molecules with opposing responses to FSH and LMB. The mRNA analysis identified 71 and 157 candidates in quadrants 1 and 9, respectively (Figure 2A and Supplementary Table S3). Candidate miRNAs and circRNAs were selected from the same quadrants (Figure 2B,C and Supplementary Table S3).

GO classification assigned candidate mRNAs to categories including cellular processes and biological regulation (Figure 2D). KEGG analysis identified PI3K–Akt signaling, ECM–receptor interaction, focal adhesion and necroptosis at a nominal *p* < 0.05, providing exploratory functional context (Figure 2E). We then constructed a candidate network from predicted circRNA–miRNA and miRNA–mRNA interactions (Figure 2F). *DEAF1* and gga-miR-146a-5p formed a predicted miRNA–mRNA pair, whereas novel_circ_010489 contained a predicted gga-miR-146a-5p site. Because novel_circ_010489 originated from the *ARHGAP21* locus, it was designated circARHGAP21. circARHGAP21 met the predefined nominal-*P* discovery threshold but not the transcriptome-wide FDR threshold in the F-versus-C and L-versus-F comparisons; it was therefore prioritized as an exploratory candidate for expression analysis and construct-based functional assessment in independent donors. Among the network nodes, DEAF1 was prioritized because it is a transcription factor with experimentally characterized nuclear localization and export signals [57]. These properties supported the selection of DEAF1 as a candidate shuttling protein. The associated circARHGAP21/gga-miR-146a-5p/*DEAF1* module was therefore selected for experimental characterization.

qRT-PCR confirmed the direction of expression changes observed via sequencing. Compared with C, the F group showed increased circARHGAP21 (*p* < 0.01) and *DEAF1* (*p* < 0.05), but decreased gga-miR-146a-5p (*p* < 0.01). Compared with F, the L group showed decreased circARHGAP21 (*p* < 0.01) and *DEAF1* (*p* < 0.05), but increased gga-miR-146a-5p (*p* < 0.01; Figure 2G–I). The agreement between sequencing and qRT-PCR supported the prioritization of this module for molecular characterization and gain-of-function analyses.

### 3.3. circARHGAP21 Annotation, Relative RNase R Resistance and Subcellular Distribution of the Candidate Module

**Figure 3:**
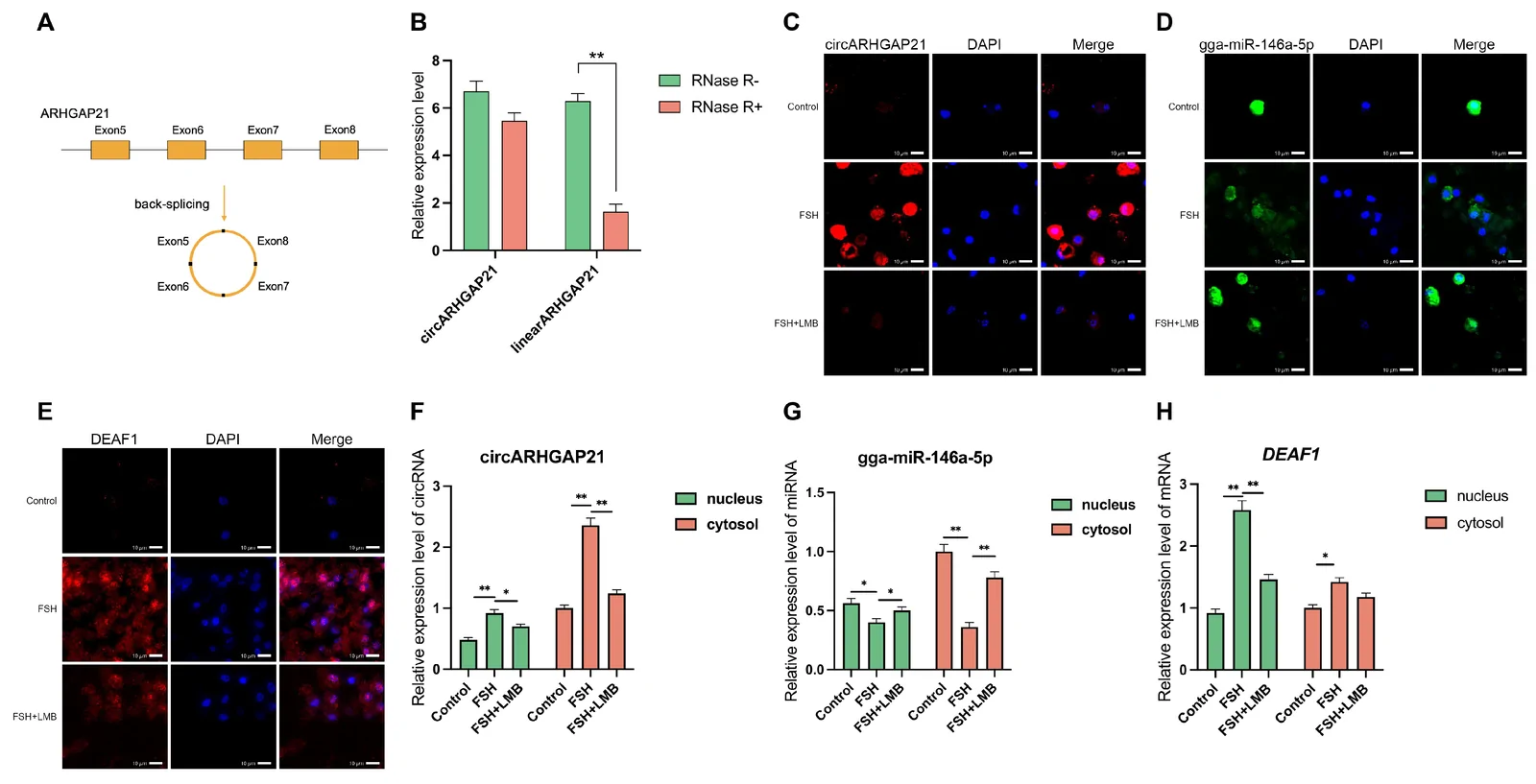
Subcellular localization and nucleocytoplasmic distribution of circARHGAP21, gga-miR-146a-5p and DEAF1. (**A**) Annotated genomic structure and predicted back-splicing pattern of circARHGAP21. (**B**) Relative expression before and after RNase R digestion in three matched biological RNA samples. (**C**,**D**) RNA-FISH localization of circARHGAP21 and gga-miR-146a-5p. (**E**) Immunofluorescence localization of DEAF1. Scale bars, 10 μm. (**F**–**H**) Relative circARHGAP21, gga-miR-146a-5p and *DEAF1* mRNA abundance in recovered nuclear and cytoplasmic fractions. Quantitative data are means ± SDs; *n* = 3 individual-hen biological samples per condition. * *p* < 0.05, ** *p* < 0.01.

Transcript annotation assigned circARHGAP21 (novel_circ_010489; 2112 nt) to a predicted back-splicing event involving *ARHGAP21* exons 5–8 (Figure 3A; Supplementary Table S5). RNase R treatment caused a highly significant reduction in linear *ARHGAP21* abundance (*p* < 0.01; Figure 3B).

RNA-FISH showed predominantly cytoplasmic circARHGAP21 and nucleocytoplasmic gga-miR-146a-5p signals (Figure 3C,D). DEAF1 immunofluorescence was mainly nuclear (Figure 3E). These images were assessed qualitatively. Following RNA fractionation, circARHGAP21, gga-miR-146a-5p and *DEAF1* mRNA were quantified separately in the recovered nuclear and cytoplasmic fractions (Figure 3F–H). FSH increased circARHGAP21 in both fractions (both *p* < 0.01), whereas LMB reduced nuclear and cytoplasmic abundance (*p* < 0.05 and *p* < 0.01, respectively). FSH decreased gga-miR-146a-5p in both fractions (*p* < 0.05 and *p* < 0.01), whereas LMB increased it (*p* < 0.05 and *p* < 0.01). FSH also increased nuclear (*p* < 0.01) and cytoplasmic (*p* < 0.05) *DEAF1* mRNA, whereas LMB reduced nuclear *DEAF1* mRNA (*p* < 0.05). Collectively, imaging described the localization of the three module components, and fractionation assays detected treatment-associated differences in RNA abundance within the recovered fractions.

### 3.4. Transfection with the oe-circARHGAP21 Construct Increases FSHR and PCNA Expression and Reduces CASP3 Expression and Apoptosis

**Figure 4:**
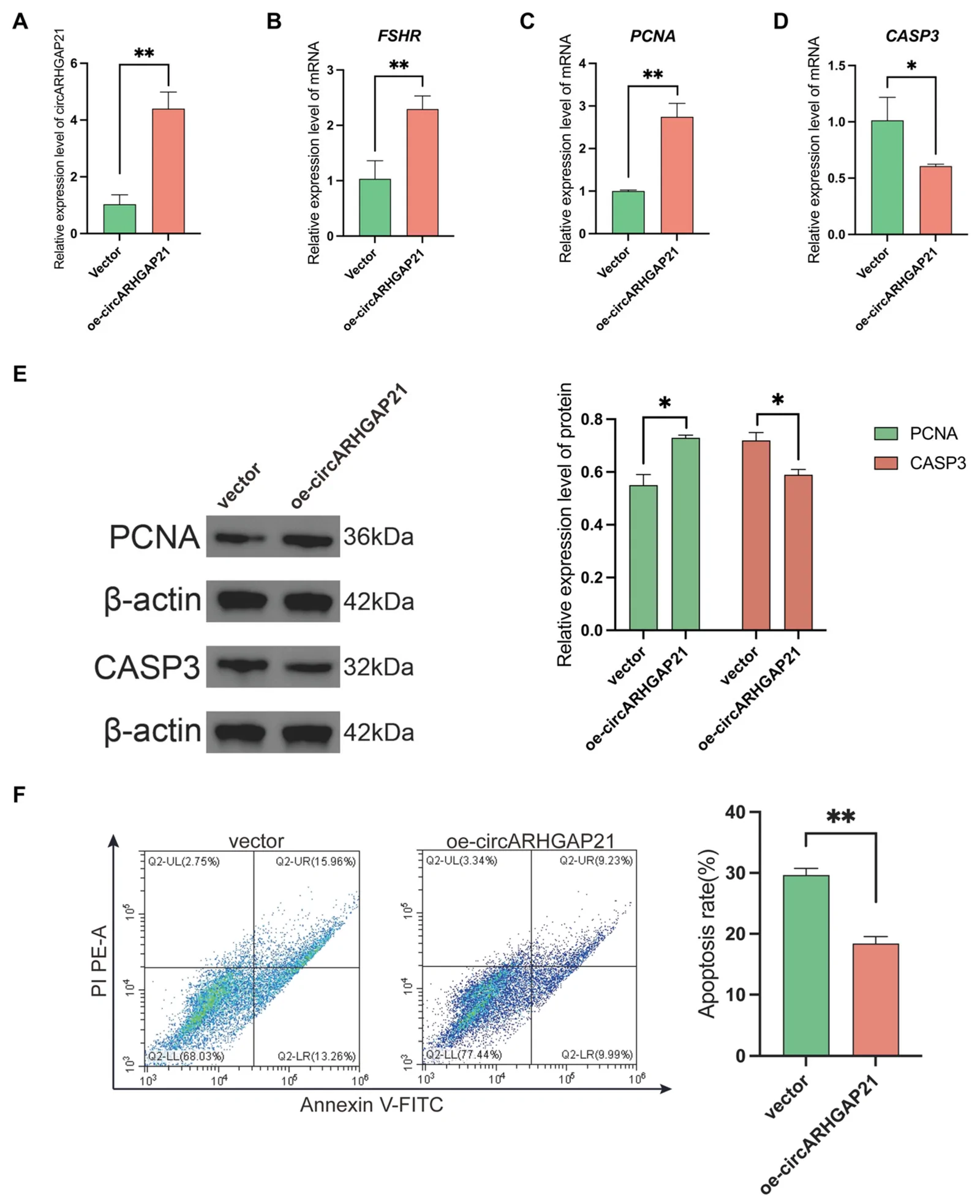
Transfection with the oe-circARHGAP21 construct increases *FSHR* and *PCNA* expression and reduces *CASP3* expression and GC apoptosis. (**A**) Junction-targeted qRT-PCR signal after oe-circARHGAP21 construct transfection. (**B**–**D**) *FSHR*, *PCNA* and *CASP3* mRNA expression. (**E**) Western blot analysis and quantification of PCNA and total CASP3 protein levels, normalized to β-actin. (**F**) Apoptosis measured via Annexin V-FITC/PI flow cytometry. Data are means ± SDs from three individual-hen biological replicates per condition. * *p* < 0.05, ** *p* < 0.01.

We next assessed molecular markers and apoptosis after transfection with the oe-circARHGAP21 construct in GCs. Junction-targeted qRT-PCR detected an increased target signal after construct transfection (*p* < 0.01; Figure 4A). Compared with empty-vector controls, transfection with the oe-circARHGAP21 construct increased *FSHR* and *PCNA* mRNA levels (both *p* < 0.01) and decreased *CASP3* mRNA (*p* < 0.05; Figure 4B–D). PCNA protein abundance increased, whereas CASP3 protein abundance decreased (both *p* < 0.05; Figure 4E). Flow cytometry confirmed a lower apoptotic fraction in cells transfected with the oe-circARHGAP21 construct (*p* < 0.01; Figure 4F). Together, these changes characterize a GC phenotype with higher *FSHR* and *PCNA* expression and reduced apoptosis.

### 3.5. The gga-miR-146a-5p Mimic Suppresses circARHGAP21 Reporter Activity and Attenuates Construct-Associated Effects

**Figure 5:**
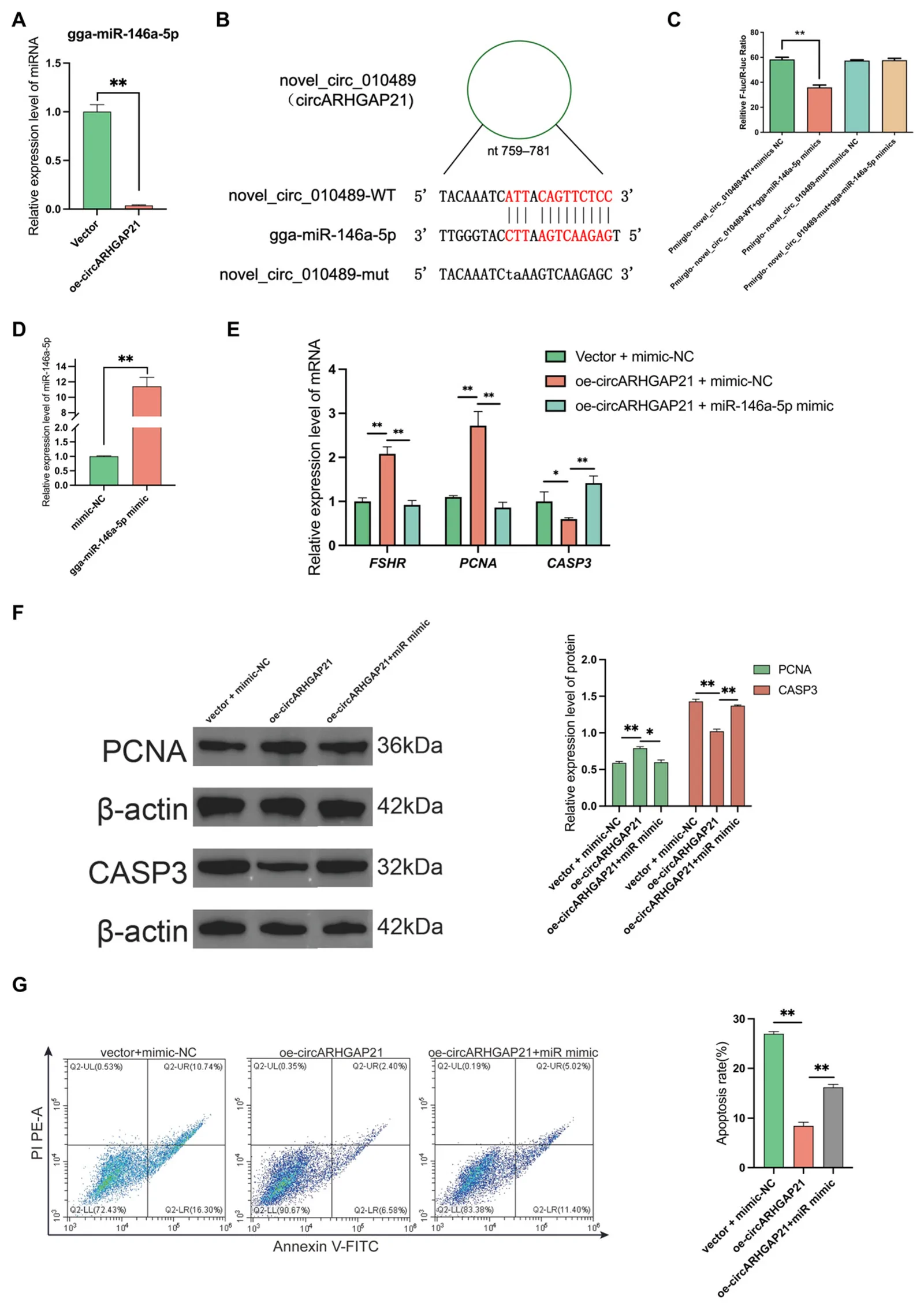
The gga-miR-146a-5p mimic suppresses circARHGAP21 reporter activity and attenuates oe-circARHGAP21-construct-associated effects. (**A**) gga-miR-146a-5p expression in empty-vector and oe-circARHGAP21 construct groups. (**B**) Predicted binding site and mutant sequence. (**C**) Wild-type and mutant circARHGAP21 reporters cotransfected with mimic NC or miRNA mimic. (**D**) gga-miR-146a-5p expression in mimic NC and mimic groups. (**E**) *FSHR*, *PCNA* and *CASP3* mRNA expression. (**F**) Western blot analysis and quantification of PCNA and total CASP3 protein levels, normalized to β-actin. (**G**) Apoptosis measured via Annexin V-FITC/PI flow cytometry. Panels (**E**–**G**) compare empty vector plus mimic NC, oe-circARHGAP21 construct plus mimic NC and oe-circARHGAP21 construct plus miRNA mimic. Reporter assays used three independent 293T experiments. GC data are means ± SDs from three individual-hen biological replicates per condition. * *p* < 0.05, ** *p* < 0.01.

Network analysis predicted a gga-miR-146a-5p-binding site within circARHGAP21. Transfection with the oe-circARHGAP21 construct reduced gga-miR-146a-5p expression (*p* < 0.01; Figure 5A). The mimic suppressed the activity of the wild-type circARHGAP21 reporter but not that of the binding-site mutant (*p* < 0.01; Figure 5B,C). qRT-PCR confirmed successful mimic transfection into GCs (*p* < 0.01; Figure 5D). Relative to mimic-negative controls, the mimic decreased *FSHR* and *PCNA* mRNAs and increased *CASP3* mRNA (all *p* < 0.01; Figure 5E). It decreased PCNA protein abundance (*p* < 0.05) and increased CASP3 protein abundance (*p* < 0.01; Figure 5F). Flow cytometry showed increased apoptosis after mimic transfection (*p* < 0.01; Figure 5G). The reporter assays support a sequence-dependent response to gga-miR-146a-5p, while mimic cotransfection attenuated the oe-circARHGAP21-construct-associated changes in marker expression and apoptosis.

### 3.6. gga-miR-146a-5p Suppresses DEAF1, Whereas DEAF1 Overexpression Increases FSHR Expression and Reduces Apoptosis

**Figure 6:**
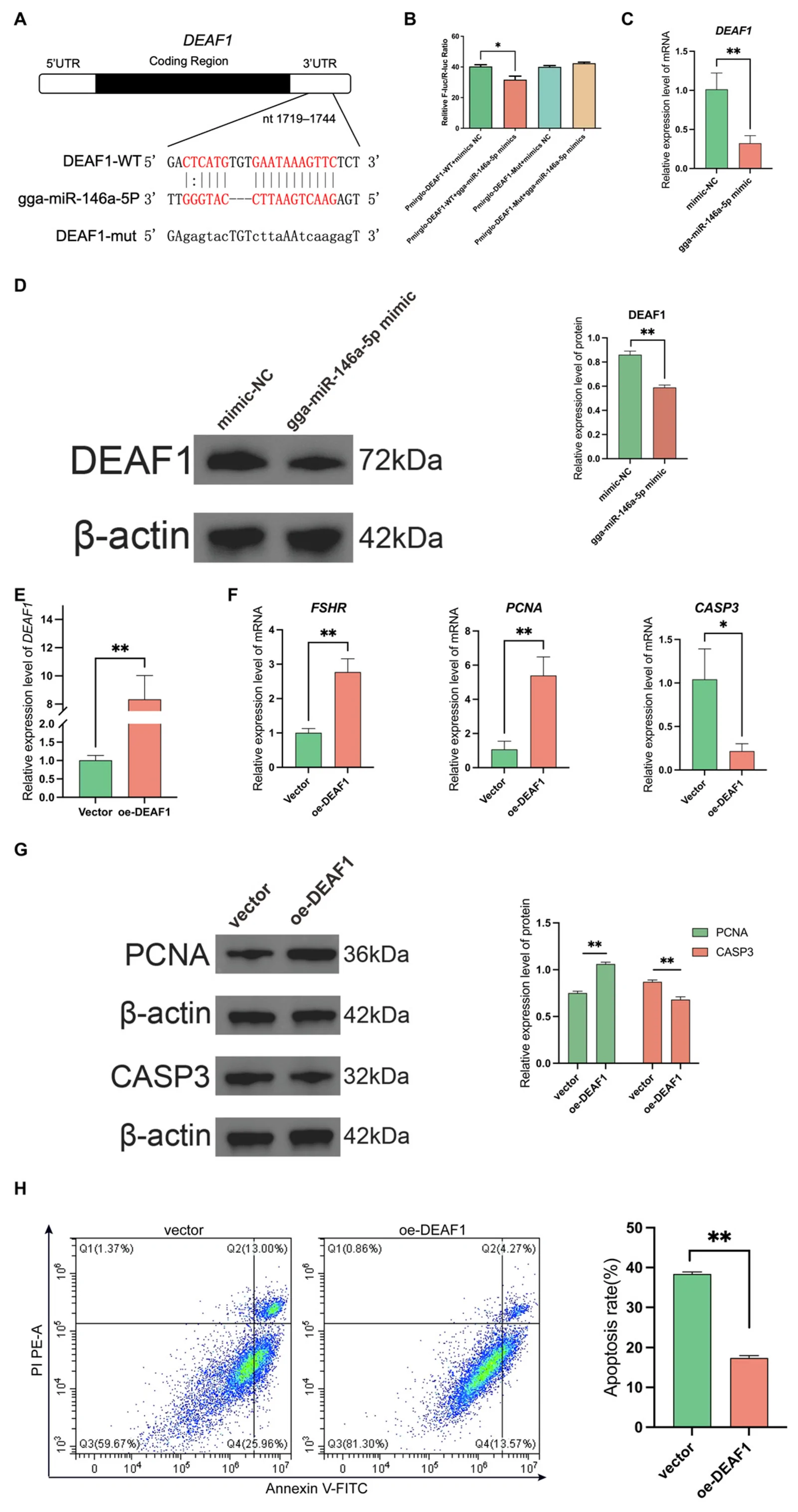
gga-miR-146a-5p suppresses *DEAF1* reporter activity and expression, whereas *DEAF1* overexpression increases *FSHR* expression and reduces apoptosis. (**A**) Predicted gga-miR-146a-5p-binding site and mutant sequence in the *DEAF1* 3′ UTR. (**B**) Dual-luciferase activity of wild-type and mutant *DEAF1* 3′ UTR reporters. (**C**,**D**) Effects of the gga-miR-146a-5p mimic on *DEAF1* mRNA and DEAF1 protein abundance. (**E**) *DEAF1* overexpression efficiency. (**F**) *FSHR*, *PCNA* and *CASP3* mRNA expression. (**G**) PCNA and total CASP3 protein abundance. Protein levels in panels (**D**,**G**) were measured via Western blotting and normalized to β-actin. (**H**) Apoptosis measured via Annexin V-FITC/PI flow cytometry. Reporter assays used three independent 293T experiments. GC data are means ± SDs from three individual-hen biological replicates per condition. * *p* < 0.05, ** *p* < 0.01.

A bioinformatic analysis predicted a gga-miR-146a-5p-binding site in the *DEAF1* 3′ UTR (Figure 6A). The mimic suppressed the activity of the wild-type *DEAF1* 3′ UTR reporter but not that of the binding-site mutant (Figure 6B). It also reduced *DEAF1* mRNA and protein abundance (both *p* < 0.01; Figure 6C,D). We next overexpressed *DEAF1* to assess its effects on GC phenotypes. qRT-PCR confirmed the increased *DEAF1* expression (*p* < 0.01; Figure 6E). *DEAF1* overexpression increased *FSHR* and *PCNA* mRNAs (both *p* < 0.01) and decreased *CASP3* mRNA (*p* < 0.05; Figure 6F). PCNA protein abundance increased, whereas CASP3 protein abundance decreased (both *p* < 0.01; Figure 6G). *DEAF1*-overexpressing cells also showed less apoptosis than empty-vector controls (*p* < 0.01; Figure 6H). Together, these data support a sequence-specific relationship between gga-miR-146a-5p and the *DEAF1* 3′ UTR and associate elevated *DEAF1* with higher *FSHR* and *PCNA* expression and reduced apoptosis.

### 3.7. LMB Treatment Alters Marker Expression and Apoptosis in GCs Transfected with the oe-circARHGAP21 Construct

**Figure 7:**
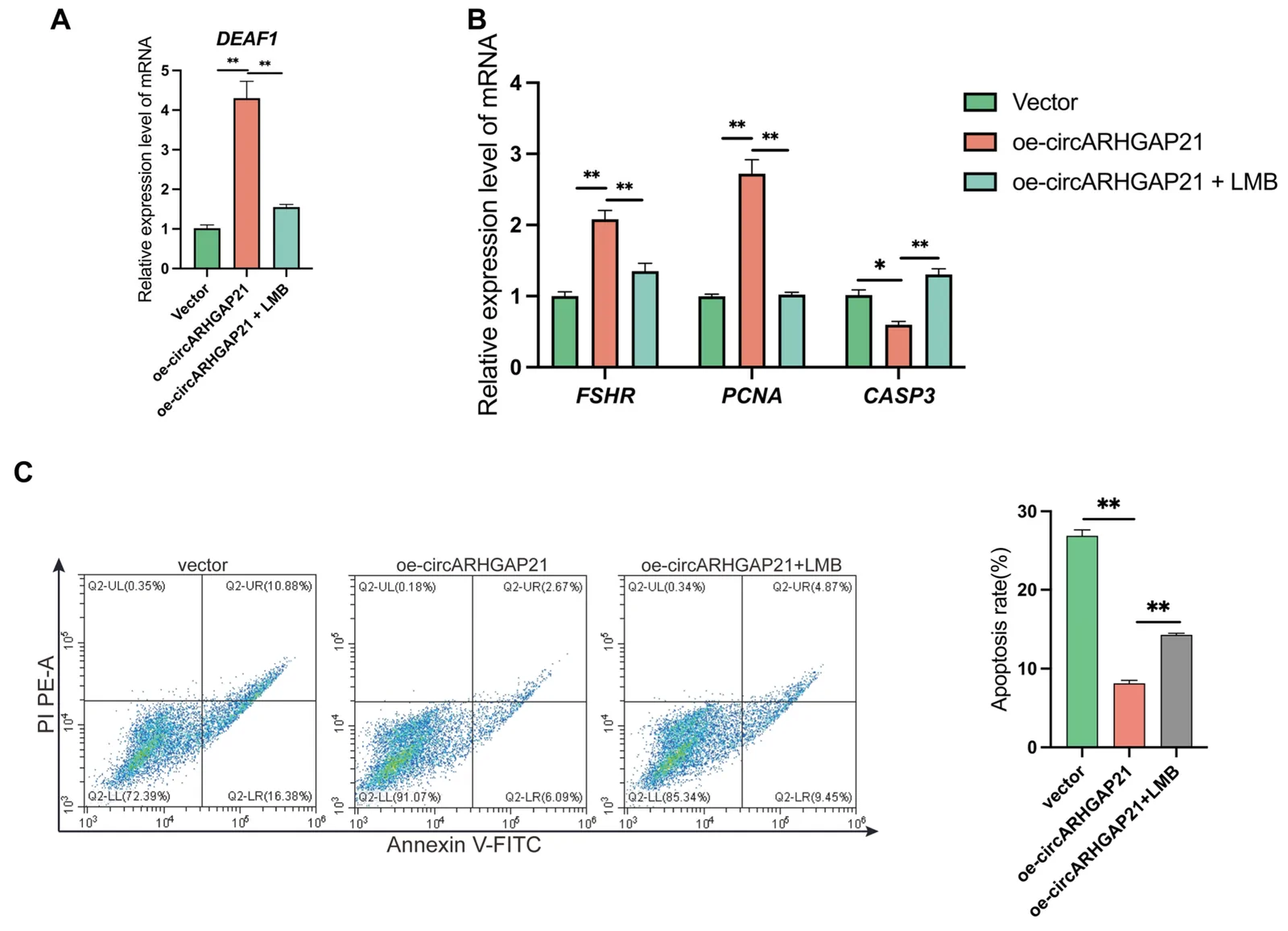
LMB treatment alters marker expression and apoptosis in GCs transfected with the oe-circARHGAP21 construct. (**A**) *DEAF1* mRNA in the indicated transfection and LMB treatment groups. (**B**) *FSHR*, *PCNA* and *CASP3* mRNA expression. (**C**) Apoptosis measured via Annexin V-FITC/PI flow cytometry. Groups were empty vector, oe-circARHGAP21 construct and oe-circARHGAP21 construct plus LMB. Data are means ± SDs from three individual-hen biological replicates per condition. * *p* < 0.05, ** *p* < 0.01.

We compared empty-vector, oe-circARHGAP21 construct and oe-circARHGAP21 construct plus LMB groups. Compared with empty-vector controls, oe-circARHGAP21 construct transfection increased *DEAF1* mRNA, whereas the construct-plus-LMB group had lower *DEAF1* mRNA than the construct-only group (both *p* < 0.01; Figure 7A). Construct transfection also increased *FSHR* and *PCNA* mRNAs (both *p* < 0.01) and decreased *CASP3* mRNA (*p* < 0.05). Compared with the construct-only group, the construct-plus-LMB group showed lower *FSHR* and *PCNA* mRNA levels and higher *CASP3* mRNA (all *p* < 0.01; Figure 7B). Flow cytometry showed a lower apoptotic fraction in the construct group than in empty-vector controls and a higher apoptotic fraction in the construct-plus-LMB group than in the construct-only group (both *p* < 0.01; Figure 7C). These comparisons describe expression and apoptosis differences among the tested treatment conditions.

## 4. Discussion

Follicle selection requires GC differentiation and survival within the hen ovary [1,2,3,4,58,59,60]. Within the established framework of FSH/FSHR signaling [5,6,7,8,9,10], the circARHGAP21/gga-miR-146a-5p/*DEAF1* module links FSH-responsive RNA expression to GC survival. Integrating transcriptomic screening with reporter and cellular perturbation experiments provides a functional basis for investigating this relationship in prehierarchical follicles.

Nucleocytoplasmic transport provides a spatial layer of gene regulation, with CRM1/XPO1 serving as a major protein-export pathway [14,15,16,17,18,19,61,62,63]. DEAF1’s reported localization, LMB-sensitive export signal and cellular functions make it a biologically relevant candidate in this context [57,64,65,66]. The FSH/LMB screen identified RNAs responsive to FSH and to LMB addition in an FSH-treated background, providing a pharmacological context for candidate selection. The reported properties of CRM1 and DEAF1 motivate this approach but do not establish CRM1-dependent regulation of the proposed module in these experiments.

Circular RNAs can participate in RNA-mediated regulation through several molecular interactions [22,23,24,25,26,27,28,37,38,39,40,41]. The cytoplasmic localization and sequence-dependent reporter response of circARHGAP21 are consistent with an miRNA-related regulatory role. The reduction in total miRNA abundance could reflect altered production or turnover, as well as indirect regulation, and does not by itself establish endogenous miRNA competition. The proposed module therefore provides a framework for testing how circARHGAP21 influences gga-miR-146a-5p activity and *DEAF1* expression.

Ovarian miRNAs regulate GC phenotypes [30,31,32,33], and miR-146a-5p has been associated with AKT signaling and reproductive functions in chicken GCs [34,35,36]. The identification of *DEAF1* as a target links this miRNA to an additional candidate effector in GCs. Previous circRNA studies in avian and mammalian GCs have examined developmental, environmental and stress-related regulation [29,67,68,69,70]. In chicken GCs, circRPS19/miR-218-5p/*INHBB* was linked to proliferation and steroidogenesis [71], whereas gga-miR-30b-5p/*NR5A2* was implicated in apoptosis in the atrophic ovaries of broody chickens [72]. In another atresia-associated network, circRALGPS2 was reported to promote GC apoptosis and autophagy through the miR-200a-3p/TGFβ2/SMAD pathway [73]. In contrast to this pro-apoptotic association, transfection with the oe-circARHGAP21 construct was associated with reduced apoptosis in our study. The proposed module is distinguished by its *DEAF1* target and FSH/LMB-guided identification, linking hormone-responsive RNA expression to GC survival within an exploratory regulatory model.

GC survival and the acquisition of FSH responsiveness are complementary aspects of follicular development. *FSHR* is a molecular marker of follicle selection: elevated expression was detected in a presumptively selected follicle within the hen 6–8 mm cohort [11]. BMP4 also maintained *FSHR* expression and enabled FSH-induced cAMP and progesterone production in prehierarchical GCs [74]. Together, these findings link *FSHR* expression to the acquisition of gonadotropin responsiveness and support interpreting our *FSHR* changes as selection-associated molecular responses. Annexin V-FITC/PI measurements provide the functional apoptosis endpoint, while *FSHR*, PCNA and total CASP3 expression offer complementary molecular context. PCNA abundance is not a direct proliferation measurement, and total CASP3 abundance does not measure caspase-3 activation.

The study design defines the scope of the proposed model. The functional transfection results are attributed to the oe-circARHGAP21 construct rather than specifically to its circular RNA product. Junction sequencing and direct characterization of the construct-derived RNA species are required to resolve circular and linear contributions [75]; endogenous necessity, miRNA competition and *DEAF1*-dependent mediation also require direct testing. DEAF1 protein imaging and fractionated RNA measurements describe localization and recovered-fraction abundance; without compartment-purity controls, these measurements do not establish quantitative redistribution or transport between compartments. Without LMB-only and empty-vector-plus-LMB controls, the observed LMB responses cannot distinguish general drug effects from interactions with FSH or the oe-circARHGAP21 construct. Treatment-specific reference gene stability also warrants caution; three biological replicates per condition limit precision and the power to assess distributional assumptions. Within this scope, the expression, reporter and cellular findings support further investigations of the candidate module in FSH-responsive RNA regulation and GC survival.

## 5. Conclusions

Integrated transcriptomic screening and cellular perturbation experiments identified a candidate circARHGAP21/gga-miR-146a-5p/*DEAF1* module associated with FSH-regulated expression in chicken GCs. Transfection with the oe-circARHGAP21 construct increased *FSHR* and *PCNA* expression and reduced apoptosis, while miRNA-mimic cotransfection and *DEAF1* overexpression provided complementary support for the proposed regulatory model. The LMB comparisons describe drug-associated expression and phenotypic changes in the tested treatment backgrounds. Together, these findings identify a candidate network linking FSH-responsive RNA expression to GC survival and follicle-selection-related molecular responses.

## Figures and Tables

**Table 1:** Primer sequences used in this study.

Target	Forward Primer (5′→3′)	Reverse Primer (5′→3′)
circARHGAP21	AGAGAGGAAAGCATCAGGTTCC	GTCTTCCCAATAACGCTTTCTCC
linear*ARHGAP21*	AGGCCAGGTGATCCAAATGG	ATGTGCTGGTGTGAGGATGG
gga-miR-146a-5p	GAGGTGAGAACTGAATTCCA	AGTGCAGGGTCCGAGGTATT
*DEAF1*	GCAGACAACGTGTTCACCAC	GCCGTCTGTGTGAACCACTA
*FSHR*	GCACCTTCCAAGCCTCAGAT	AGAACTCAGGCCCATGAACG
*PCNA*	CCTGTGCAAAAGACGGTGTG	CAGCTGGACTGGCTCATTCA
*CASP3*	ATCTCGTTCAGGCACGGATG	AAACTGCTTCGCTTGCTGTG
*GAPDH*	CCAGAACATCATCCCAGCGTC	ACGGCAGGTCAGGTCAACAA
*U6*	CTTGCTTCGGCAGCACA	AACGCTTCACGAATTTGCGT
*β-actin*	GTCCACCGCAAATGCTTCTAA	TGCGCATTTATGGGTTTTGTT

**Table 2:** RNA fluorescence in situ hybridization probes and hybridization conditions.

Target RNA	Probe Name	Probe Sequence (5′–3′)	Pretreatment	Conc. (nM)	Temp. (°C)	Detection System
gga-miR-146a-5p	gga-miR-146a-5p-H3-488	AACCCATGGAATTCAGTTCTCA	No retrieval; 0.1% Triton X-100, 10 min, room temperature	500	40	Branch probe 2; 488-labeled signal probe 2
circARHGAP21 (novel_circ_010489)	novel_circ_010489-H2-Cy3	TTATAATTCTGTCACCAATAAATGGAATAGA	No retrieval; 0.1% Triton X-100, 10 min, room temperature	500	40	Branch probe 1; Cy3-labeled signal probe 1

**Table 3:** Antibodies used for Western blotting.

Antibody	Dilution	Manufacturer	Item Number
β-Actin	1:5000	Proteintech (Wuhan, China)	81115-1-RR; RRID:AB_2923704
DEAF1	1:1000	Proteintech (Wuhan, China)	18323-1-AP; RRID:AB_2261595
PCNA	1:1000	Proteintech (Wuhan, China)	10205-2-AP; RRID:AB_2160330
CASP3	1:1000	Affinity Biosciences (Cincinnati, OH, USA)	AF6311; RRID:AB_2835170
HRP-conjugated Affinipure Goat Anti-Mouse	1:5000	Proteintech Group (Chicago, IL, USA)	SA00001-1
HRP-conjugated Affinipure Goat Anti-Rabbit	1:5000	Proteintech Group (Chicago, IL, USA)	SA00001-2

## Data Availability

The RNA sequencing data generated in this study have been deposited in the NCBI Sequence Read Archive (SRA) under BioProject accession number PRJNA1531702. All other data used and analyzed during the present study are included in this published article and its Supplementary Information Files.

## Supplementary Materials

The following supporting information can be downloaded at: https://www.mdpi.com/article/10.3390/cells15211939/s1, Table S1: Long-RNA sequencing quality-control and read-alignment statistics for chicken granulosa cells; Table S2: Messenger RNA DEGs and nominal-*P* microRNA and circular RNA candidates among the control, follicle-stimulating hormone and follicle-stimulating hormone plus leptomycin B groups; Table S3: Nine-quadrant screening of messenger RNAs, microRNAs and circular RNAs showing opposing responses to follicle-stimulating hormone and leptomycin B; Table S4: RNase R digestion reaction system and matched-aliquot procedure; Table S5: circARHGAP21 sequence and transcript annotation; Table S6: GO level-2 functional classifications and KEGG pathway enrichment results underlying Figure 1 and Figure 2.

## Abbreviations

AKT	protein kinase B
ARHGAP21	Rho GTPase activating protein 21
BCA	bicinchoninic acid
BSA	bovine serum albumin
C, F and L	control, FSH-treated and FSH-plus-LMB-treated groups, respectively
cAMP	cyclic adenosine monophosphate
CASP3	caspase 3
CC BY	Creative Commons Attribution license
cDNA	complementary DNA
ceRNA	competing endogenous RNA
circARHGAP21	circular RNA derived from the ARHGAP21 locus
circRNA	circular RNA
CRM1/XPO1	chromosome region maintenance 1/exportin 1
Cy3	cyanine 3 fluorescent dye
DAPI	4′,6-diamidino-2-phenylindole
DEAF1	deformed epidermal autoregulatory factor 1
DEC(s)	differentially expressed circRNA(s)
DEG(s)	differentially expressed gene(s)
DEM(s)	differentially expressed miRNA(s)
DNA	deoxyribonucleic acid
dUTP	deoxyuridine triphosphate
ECL	enhanced chemiluminescence
ECM	extracellular matrix
EDTA	ethylenediaminetetraacetic acid
FDR	false discovery rate
FITC	fluorescein isothiocyanate
FOXO/FOXO1	forkhead box O family/forkhead box O1
FSH	follicle-stimulating hormone
FSHR	follicle-stimulating hormone receptor
GAPDH	glyceraldehyde-3-phosphate dehydrogenase
GC(s)	granulosa cell(s)
gga-miR-146a-5p	*Gallus gallus* microRNA-146a-5p
GO	Gene Ontology
H + L	heavy and light chains
HISAT2	Hierarchical Indexing for Spliced Alignment of Transcripts 2
HRP	horseradish peroxidase
IF	immunofluorescence
IgG	immunoglobulin G
KEGG	Kyoto Encyclopedia of Genes and Genomes
LMB	leptomycin B
M199	Medium 199
MARA	Ministry of Agriculture and Rural Affairs
MEM	minimum essential medium
miRNA	microRNA
MOF	Ministry of Finance
mRNA	messenger RNA
mut	mutant
NC	negative control
OD260/OD280	optical-density ratio at 260 and 280 nm
oe	overexpression
ORF	open reading frame
PBS	phosphate-buffered saline
PCA	principal component analysis
PC1/PC2	first and second principal components
PCNA	proliferating cell nuclear antigen
PCR	polymerase chain reaction
PE-A	area parameter of the phycoerythrin detection channel
PI	propidium iodide
PI3K	phosphoinositide 3-kinase
PKA	protein kinase A
PVDF	polyvinylidene fluoride
Q20/Q30	Phred quality scores of 20 and 30
qPCR	quantitative PCR
qRT-PCR	quantitative reverse-transcription PCR
RanGTP	guanosine triphosphate-bound Ran GTPase
RIPA	radioimmunoprecipitation assay buffer
RNA	ribonucleic acid
RNA-FISH	RNA fluorescence in situ hybridization
RNA-seq	RNA sequencing
RNase R	ribonuclease R
RPM	reads per million
rRNA	ribosomal RNA
SDS-PAGE	sodium dodecyl sulfate-polyacrylamide gel electrophoresis
SD	standard deviation
snRNA	small nuclear RNA
SPSS	Statistical Package for the Social Sciences
SSC	saline-sodium citrate
TBST	Tris-buffered saline containing Tween 20
Temp.	temperature
TPM	transcripts per million
tRNA	transfer RNA
U6	U6 small nuclear RNA
UTR	untranslated region
WT	wild type
